# Ketamine, a Clinically Used Anesthetic, Inhibits Vascular Smooth Muscle Cell Proliferation via PP2A-Activated PI3K/Akt/ERK Inhibition

**DOI:** 10.3390/ijms18122545

**Published:** 2017-11-27

**Authors:** Yi Chang, Jiun-Yi Li, Thanasekaran Jayakumar, Shou-Huang Hung, Wei-Cheng Lee, Manjunath Manubolu, Joen-Rong Sheu, Ming-Jen Hsu

**Affiliations:** 1 Department of Anesthesiology, Shin Kong Wu Ho-Su Memorial Hospital, No. 95, Wenchang Rd., Taipei 111, Taiwan; m004003@ms.skh.org.tw; 2 School of Medicine, Fu-Jen Catholic University, No. 510, Zhongzheng Rd., Xinzhuang Dist, New Taipei City 242, Taiwan; 3 Department of Pharmacology and Graduate Institute of Medical Sciences, College of Medicine, Taipei Medical University, No. 250, Wu-Hsing St., Taipei 110, Taiwan; JYL5891@ms2.mmh.org.tw (J.-Y.L.); jayakumar@tmu.edu.tw (T.J.); b8301033@tmu.edu.tw (S.-H.H.); m120097037@tmu.edu.tw (W.-C.L.); 4 Department of Cardiovascular Surgery, Mackay Memorial Hospital, and Mackay Medical College, Taipei 104, Taiwan; 5 Department of Evolution, Ecology and Organismal Biology, Ohio State University, 1314 Kinnear Rd, Columbus, OH 43212, USA; manubolu.1@osu.edu

**Keywords:** ketamine, VSMC, PDGF-BB, Akt, ERK1/2, PP2A, *pp2a* siRNA

## Abstract

Abnormal proliferation of vascular smooth muscle cells (VSMCs) gives rise to major pathological processes involved in the development of cardiovascular diseases. The use of anti-proliferative agents for VSMCs offers potential for the treatment of vascular disorders. Intravenous anesthetics are firmly established to have direct effects on VSMCs, resulting in modulation of blood pressure. Ketamine has been used for many years in the intensive care unit (ICU) for sedation, and has recently been considered for adjunctive therapy. In the present study, we investigated the effects of ketamine on platelet-derived growth factor BB (PDGF-BB)-induced VSMC proliferation and the associated mechanism. Ketamine concentration-dependently inhibited PDGF-BB-induced VSMC proliferation without cytotoxicity, and phosphatidylinositol 3-kinase (PI3K) and extracellular signal-regulated protein kinase (ERK) inhibitors, LY294002 and PD98059, respectively, have similar inhibitory effects. Ketamine was shown to attenuate PI3K, Akt, and ERK1/2 phosphorylation induced by PDGF-BB. Okadaic acid, a selective protein phosphatase 2A (PP2A) inhibitor, significantly reversed ketamine-mediated PDGF-BB-induced PI3K, Akt, and ERK1/2 phosphorylation; a transfected protein phosphatse 2a (*pp2a*) siRNA reversed Akt and ERK1/2 phosphorylation; and 3-*O*-Methyl-sphingomyeline (3-OME), an inhibitor of sphingomyelinase, also significantly reversed ERK1/2 phosphorylation. Moreover, ketamine alone significantly inhibited tyrosine phosphorylation and demethylation of PP2A in a concentration-dependent manner. In addition, the *pp2a* siRNA potently reversed the ketamine-activated catalytic subunit (PP2A-C) of PP2A. These results provide evidence of an anti-proliferating effect of ketamine in VSMCs, showing activation of PP2A blocks PI3K, Akt, and ERK phosphorylation that subsequently inhibits the proliferation of VSMCs. Thus, ketamine may be considered a potential effective therapeutic agent for reducing atherosclerotic process by blocking the proliferation of VSMCs.

## 1. Introduction

Cardiovascular diseases (CVDs) are the most common causes of death. Atherosclerosis, a chronic inflammatory disease of the vessel wall, is the principal underlying pathology of CVD [1]. Atherosclerotic plaques are intricate injuries in which the repair of tissue damage is associated with vascular smooth muscle cell (VSMC) proliferation [2]. In the development of vascular disease, VSMCs contribute a vital role; the abnormal proliferation of VSMCs has a central role in the progress of atherosclerosis and restenosis [3]. Different cytokines and growth factors, such as platelet-derived growth factor (PDGF), tumor necrosis factor (TNF)-α and transforming growth factor (TGF)-β induce the proliferation of VSMC [4]. PDGF is a major growth factor and is known to contribute to the development of atherosclerosis through the induction of abnormal VSMC phenotypes [5]. Thus, the controlling mechanism of PDGF-BB signaling is one of the crucial pharmacological approaches for the prevention of atherosclerosis via inhibition of VSMC proliferation. Various intracellular signaling molecules, such as extracellular signal-regulated protein kinase (ERK), c-Jun N-terminal kinase (JNK), p38 mitogen-activated protein kinase (MAPK), phosphatidylinositol 3-kinase (PI3K), and protein kinase C (PKC) are associated with PDGF-BB-induced rat aortic VSMC migration [6]. Among the four families of serine/threonine protein phosphatases (PP1, PP2A, PP2B and PP2C), PP2A is a multifaceted molecule, comprising three different subunits, such as PP2A-C, PP2A-A, and PP2A-B [7]. The regulation of expression and function of specific PP2A in VSMCs remain largely unknown. 

In view of the effects of anesthetics on VSMC migration, a previous study showed that dexmedetomidine, a potent and highly selective α-2 adrenoceptor agonist, increased primary rat VSMC cell migration [8]. Intravenous anesthetics have been decisively established to have direct effects on VSMCs, with modulation of blood pressure ensuing. Ketamine, a well-known anesthetic agent, has gained substantial interest as part of the multimodal approach toward acute pain treatment [9]. It has been used for many years in the intensive care unit (ICU) setting for sedation, and has recently been considered for adjunctive therapy [10]. Ketamine induces vasorelaxation via reducing the intracellular Ca^2+^ concentration ([Ca^2+^]i) and myofilament Ca^2+^ sensitivity [11]. As an *N*-methyl-d-aspartate (NMDA) receptor antagonist [12], ketamine can act as a pain reliever by hindering the NMDA receptors coupled in nociceptive and inflammatory pain transmission [13]. Considering ketamine’s analgesic properties, it may lead to the development of clinical trials to evaluate the drug’s capacity to alleviate various pain syndromes, including cancer, neuropathic, refractory chronic, and acute pain. However, the effects of ketamine on VSMC proliferation have not yet been clarified. Thus, the aim of this study was to elucidate the effects of ketamine on PDGF-BB-stimulated VSMC proliferation, as well as its mechanism.

## 2. Results and Discussion

### 2.1. Results

#### 2.1.1. Effects of Ketamine on Platelet-Derived Growth Factor BB (PDGF-BB)-Induced Toxicity and Proliferation of Vascular Smooth Muscle Cells (VSMCs)

**Figure 1:**
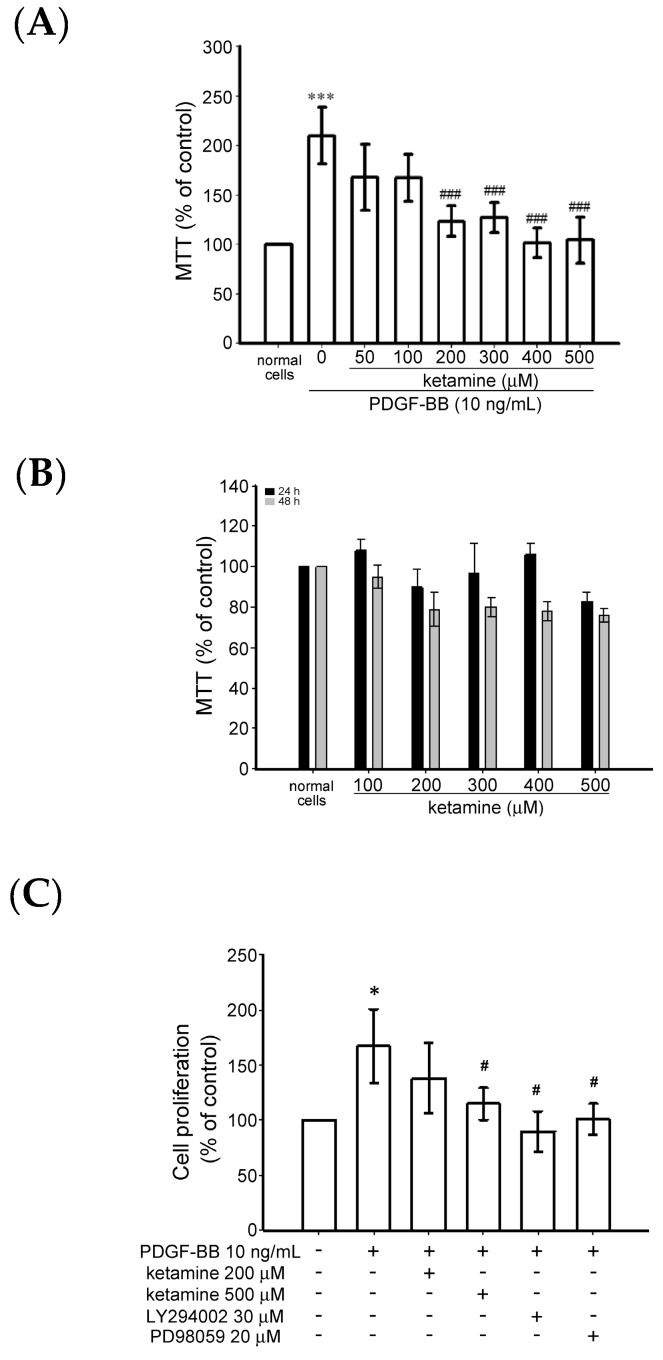
Effects of ketamine on platelet-derived growth factor BB (PDGF-BB)-induced cytotoxicity and proliferation of vascular smooth muscle cells (VSMCs). (**A**,**B**) Cytotoxicity and (**C**) proliferation were observed via MTT [3-(4,5-dimethylthiazol-2-yl)-2,5-diphenyltetrazolium bromide) tetrazolium] and BrdU (bromodeoxyuridine) assay, respectively. VSMCs were pre-cultured in serum-free medium in the presence or absence of ketamine (50–500 μM) for 30 min, and then stimulated with 10 ng/mL PDGF-BB for a further 24 h (*n* = 4). (**B**) VSMCs were treated with 100–500 μM of ketamine in serum-free medium for 24 (closed column) and 48 (shaded column) h (*n* = 4). (**C**) VSMCs were treated with 200 and 500 μM of ketamine or 30 μM LY294002 (phosphatidylinositol 3-kinase (PI3K) inhibitor) and 20 μM PD98059 extracellular signal-regulated protein kinase (ERK1/2 inhibitor) in serum-free medium for 30 min and then stimulated with 10 ng/mL PDGF-BB for 24 h. Cytotoxicity and cell proliferation were measured at 550 and 370 nm, respectively. Data are presented as means ± standard errors of the means (*n* = 4). *** *p* < 0.001 and * *p* < 0.05, compared with the normal cells; ^###^ *p <* 0.001 and ^#^ *p <* 0.05, compared with the PDGF-BB-treated cells.

The effect of ketamine on the PDGF-BB-induced cytotoxicity of VSMC was examined using the MTT [3-(4,5-dimethylthiazol-2-yl)-2,5-diphenyltetrazolium bromide) tetrazolium] assay. VSMCs were pretreated with ketamine (50–500 µM) in a serum-free medium for 30 min and then stimulated with PDGF-BB (10 ng/mL) for 24 h. PDGF-BB treatment significantly increased the toxicity of the VSMCs up to 1.2 folds, compared with the untreated cells (Figure 1A). PDGF-BB-induced cytotoxicity was significantly reduced by ketamine (50–500 µM) treatment. (Figure 1A). Moreover, without PDGF-BB, ketamine treatment alone (100–500 µM) for 24 and 48 h did not affect the viability of the VSMCs (Figure 1B). 

In addition, bromodeoxyuridine (BrdU) assay revealed that ketamine at a maximum of 500 µM significantly (*p* < 0.05) suppressed the PDGF-BB-induced cell proliferation (Figure 1C). PDGF-BB-induced cell proliferation was also inhibited by PI3K inhibitor LY294002 (30 µM) and ERK inhibitor PD98059 (20 µM). These results indicate that ketamine appeared to inhibit PDGF-BB-induced VSMC proliferation without cytotoxicity and this suppressive effect may be attributed to the PI3K and ERK1/2 signaling pathways. 

#### 2.1.2. Ketamine Suppressed PDGF-BB-Induced Phosphatidylinositol 3-Kinase (PI3K) and Akt Signaling Pathway

**Figure 2:**
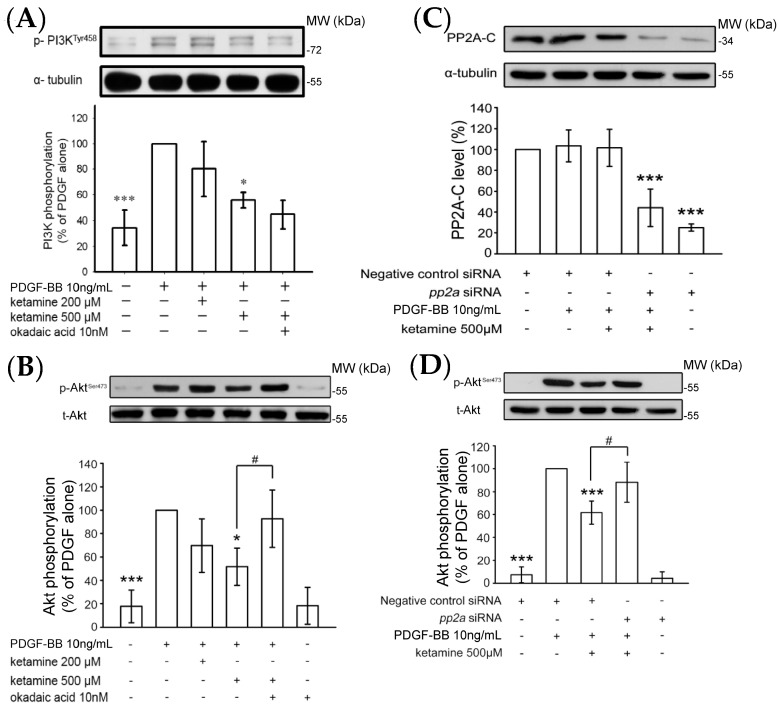
Effects of ketamine, okadaic acid, and protein phosphatase 2a (*pp2a*) siRNA on the PDGF-BB-induced phosphorylation of PI3K and Akt in VSMCs. (**A**,**B**) VSMCs were pretreated with 10 nM okadaic acid and then treated with ketamine (200 and 500 μM), followed by the addition of PDGF-BB (10 ng/mL). (**A**) PI3K (85 kDa) and (**B**) Akt (60 kDa) phosphorylation were determined as described in Materials and Methods. (**C**) The VSMCs were transiently transfected with scrambled siRNA (control) or *pp2a* siRNA, and then treated with ketamine (500 μM), followed by the addition of PDGF-BB (10 ng/mL). The PP2A catalytic subunit (PP2A-C, 36 kDa) was analyzed by immunoblotting assay using anti-protein phosphatase 2A-C. (**D**) VSMCs were transiently transfected with scrambled siRNA (control) or *pp2a* siRNA, and then treated with ketamine (500 μM), followed by the addition of PDGF-BB (10 ng/mL) and Akt phosphorylation was determined as described in Materials and Methods. Data are presented as means ± standard errors of the means (*n* = 4). * *p* < 0.05 and *** *p* < 0.001, compared with the PDGF-BB group. ^#^ *p* < 0.05 compared with the PDGF-BB + ketamine treated group. MW = Molecular weight of the protein marker.

The PI3K/Akt pathway displays an imperative role in cell survival and growth in reaction to a multiple of agents, including cytokines, growth factors, and hormones [14]. To investigate the mechanisms of the anti-proliferative effects of ketamine, we examined whether ketamine affected the phosphorylation of PI3K p85 (Tyr458) and Akt (Ser473) in PDGF-BB-stimulated VSMCs. First of all, VSMCs were stimulated with PDGF-BB for 2, 10, and 30 min without ketamine pretreatment, and it was found that PDGF-BB stimulation for 10 min markedly increased the phosphorylation of PI3K and Akt in VSMCs. Therefore, an inducible 10 min PDGF-BB stimulation was employed for the subsequent ketamine treatment. Ketamine at a 500 µmol concentration significantly inhibited PI3K and Akt phosphorylation induced by PDGF-BB for 10 min in VSMCs (Figure 2A,B). 

The blockade of PDGF-BB-induced Akt phosphorylation by ketamine was almost absolutely reversed by okadaic acid, a specific inhibitor of PP2A, while that of PI3K was not altered. To confirm that the effects of ketamine in PDGF-BB-stimulated VSMCs are mediated by PP2A, we transfected VSMCs with protein phosphatase 2a (*pp2a*) siRNA. The results showed that *pp2a* siRNA suppressed the basal level of the PP2A catalytic subunit (PP2A-C) (Figure 2C). Moreover, transfection of VSMCs with *pp2a* siRNA also significantly recovered from the ketamine-induced inhibition of Akt phosphorylation in PDGF-BB-stimulated VSMCs (Figure 2C). These results suggest that PP2A may be precisely accountable for ketamine-mediated inhibition of PDGF-BB-induced Akt phosphorylation in VSMCs.

#### 2.1.3. Effects of Ketamine, Okadaic Acid, 3-*O*-Methyl-Sphingomyeline (3-OME), and *pp2a* SiRNA on the PDGF-BB-Induced Phosphorylation of ERK1/2 in VSMCs

**Figure 3:**
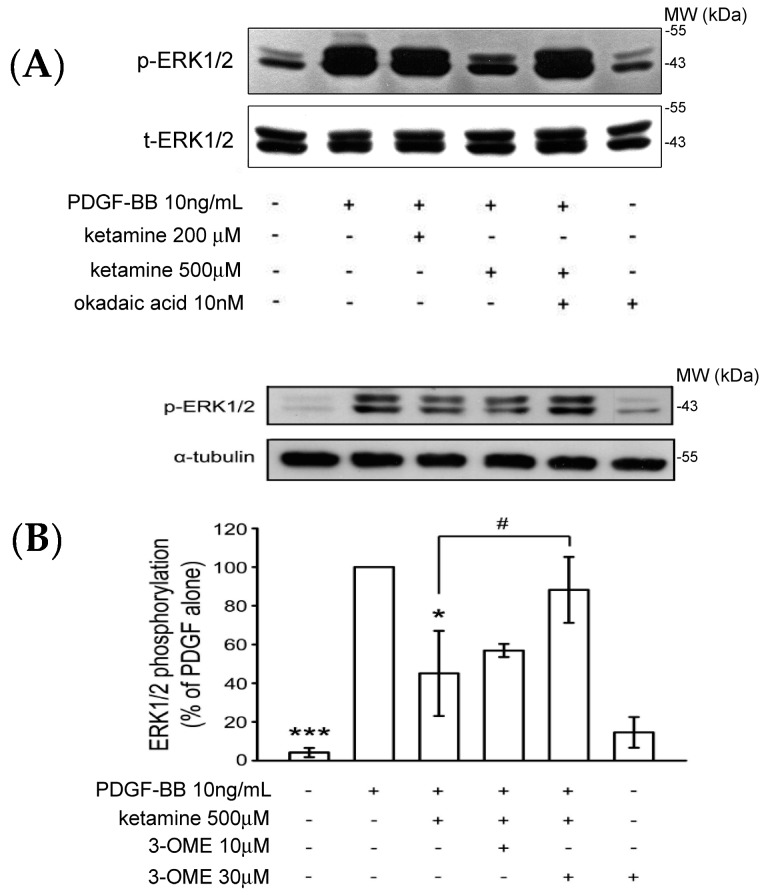

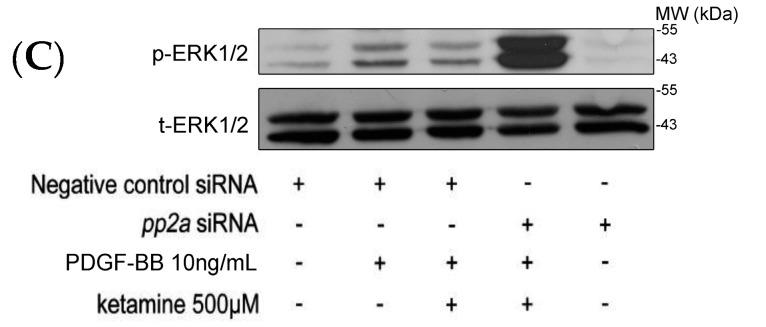
Effects of ketamine, okadaic acid, 3-*O*-methyl-sphingomyeline (3-OME) and *pp2a* siRNA on the PDGF-BB-induced phosphorylation of ERK1/2 in VSMCs. (**A**,**B**) VSMCs were pretreated with 10 nM okadaic acid and 10–30 μM 3-OME, then treated with ketamine (**A**) 200 and 500 μM and (**B**) 500 μM, followed by the addition of PDGF-BB (10 ng/mL). (**C**) VSMCs were pretreated with 10 nmol okadaic acid and 10–30 μM 3-OME, then treated with ketamine (**A**) 200 and 500 μM and (**B**) 500 μM, followed by the addition of PDGF-BB (10 ng/mL). (**C**) VSMCs were transiently transfected with scrambled siRNA (control) or *pp2a* siRNA, and then treated with ketamine (500 μM), followed by the addition of PDGF-BB (10 ng/mL). (**A**–**C**) ERK1/2 (44/42 kDa) phosphorylation was determined as described in Materials and Methods. Data are presented as means ± standard errors of the means (*n* = 4). * *p* < 0.05, *** *p* < 0.001, compared with the PDGF-BB group. ^#^ *p* < 0.05 compared with the PDGF-BB + ketamine treated group. MW = Molecular weight of the protein marker.

A previous study showed that the Akt and ERK1/2 signaling pathway plays an important role in the proliferation and migration of PDGF-BB stimulated VSMCs [15]. We examined whether ketamine affected the levels of phosphorylated ERK1/2 as it did those of Akt in PDGF-BB-stimulated VSMCs. As shown in Figure 3, PDGF-BB increased the levels of phosphorylated ERK1/2, which were inhibited by treatment of ketamine at 500 µM. The inhibition of PDGF-BB-induced ERK1/2 phosphorylation by ketamine was exactly reversed by okadaic acid and 3-OME (sphingomyelinase inhibitor) (Figure 3A,B). Moreover, transfection of *pp2a* siRNA more profoundly reversed the ketamine-inhibited ERK1/2 phosphorylation (Figure 3C). Thus, these results demonstrated that ketamine inhibited the PDGF-BB stimulated ERK1/2 phosphorylation via PP2A (a member of the ceramide-activated protein phosphatase family, known to be activated by ceramide) activation.

#### 2.1.4. Influence of Ketamine and *pp2a*-SiRNA on Phosphorylated PP2A (Tyr307) and Methylated PP2A (Leu309) in VSMCs

**Figure 4:**
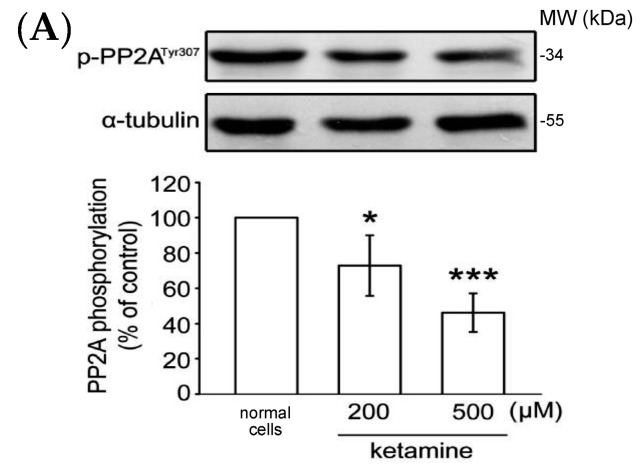

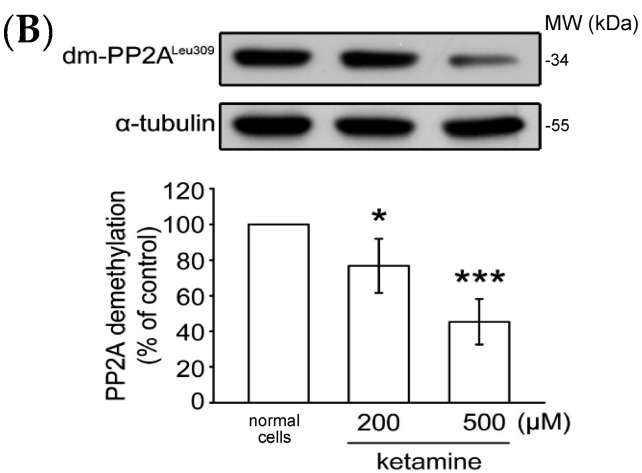
Influence of ketamine on (**A**) phosphorylated PP2A (Tyr307, 36 kDa) and (**B**) demethylated (dm-) PP2A (Leu309, 36 kDa) in VSMCs. (**A**,**B**) VSMCs were treated with DMSO (normal cells) and then treated with 200 and 500 μM ketamine for 20 min. The phosphorylation (**A**) and methylation (**B**) of PP2A were analyzed by immunoblotting assay using anti-phospho-protein phosphatase 2A (p-PP2A) and anti-demethyl-PP2A (dm-PP2A) antibodies. Data are presented as means ± standard errors of the means (*n* = 4). * *p* < 0.05, *** *p* < 0.001 compared with the normal cells. MW = Molecular weight of the protein marker.

As previously reported, PP2A Tyr307 phosphorylation or Leu309 demethylation negatively regulate PP2A activity [16]. We thus determined whether the status of PP2A Tyr307 phosphorylation or Leu309 demethylation is alerted in the presence of ketamine. As shown in Figure 4A, ketamine at 200 and 500 µM, concentration-dependently reduced PP2A Tyr307 phosphorylation when compared to normal cells in VSMCs. Ketamine also reduced PP2A Leu309 demethylation in a concentration-dependent manner (Figure 4B). Together, these findings suggest that PP2A may play an important role in the inhibitory effects of ketamine in PDGF-BB-stimulated rat VSMC proliferation.

### 2.2. Discussion

VSMC proliferation plays an important role in the development of atherosclerosis lesions [17], and, hence, the inhibition of VSMC proliferation might be a chief beneficial approach for atherosclerosis-related diseases [18]. In the present study, we identified ketamine, a noncompetitive antagonist of the NMDA receptor, as a potent antiproliferative agent for VSMCs. A literature hunt indicated that ketamine has analgesic and amnesic effects, and has potential anti-depressant properties [19,20]. A number of studies have found that high-dose ketamine treatment induces aberrantly high levels of neuroapoptosis in rodents and nonhuman primates [21]. To date, there is no study for the effect of ketamine on cellular proliferation. Therefore, for the first time, this study examined the effect of ketamine on rat vascular smooth muscle cell (VSMC) proliferation. Our data clearly demonstrated that ketamine significantly inhibited PDGF-BB-induced VSMC proliferation via suppression of the cell survival signaling pathways, such as PI3K, Akt, and ERK. This study also demonstrated that PP2A plays a major role in ketamine’s inhibitory effects on VSMC proliferation. This study concludes that ketamine may be a potential candidate for the prevention and treatment of vascular inflammatory diseases. 

Among the mitogen-activated protein kinase (MAPK) family, ERK1/2 has been majorly involved in the growth of various cell types [22]. Akt, a serine/threonine protein kinase found to be activated via the PI3K pathway, has been involved in VSMC proliferation, cell cycle progression, and cell survival [23]. The current study shows that ketamine is able to suppress the increased proliferation in the presence of PDGF-BB, and this inhibitory effect was boosted by combined treatment with PI3K inhibitor LY294002 and ERK inhibitor PD98059. These results indicate that the Akt and ERK pathway may be involved in the ketamine-mediated inhibition of VSMC proliferation. PI3K/Akt is recognized as one of the major signaling molecules for cell proliferation and survival facilitated by extracellular stimuli [24]. Substances for suppression of the PI3K/Akt pathway have used extensively in the treatment of hypertension and angina, and display a range of biological properties in the cardiovascular system. A previous study found that PI3K/Akt is greatly expressed in human as well as murine atherosclerotic lesions, and PI3K inhibitor AS605240 considerably reduced these lesions in apolipoprotein E (*Apo-E*)-null mice [25]. These authors have also found that this PI3K inhibitor was effective on advanced atherosclerotic lesions of low-density lipoprotein (LDL)-receptor-deficient mice, and suggested that PI3K might be a promising target for the treatment of atherosclerosis [25]. Moreover, topotecan, a water-soluble camptothecin analog, is reported to downregulate the PI3K/Akt signaling pathway for the inhibition of vascular endothelial growth factor (VEGF)- and basic fibroblast growth factor (bEGF)-induced vascular endothelial cell migration [26]. In some essential biologic processes, the PI3K/Akt inhibitors have been used extensively as pharmaceutical tools and accompanying signaling pathways [27]. Hence, this perception into the role of the PI3K/Akt in human diseases may offer a wide spectrum of therapeutic strategies. In harmony with the above evidence, this study found that increased PI3K, Akt, and ERK1/2 phosphorylation stimulated by PDGF-BB was potently inhibited by ketamine. These results indicate that inhibiting PDGF-BB-induced activation of the PI3K, Akt, and ERK1/2 signaling pathway may have contributed to the inhibition of VSMC proliferation exerted by ketamine. 

PP2A is one of the major Ser/Thr phosphatases associated with the regulation of various cellular processes [28]. PP2A controls numerous cell-signaling pathways by triggering dephosphorylation of various signaling proteins. A previous study has shown that PP2A can directly dephosphorylate Akt and ERK [29]. A report indicated that treatment with PP2A inhibitor okadaic acid (OA) not only inhibited PP2A activation, but also triggered Akt and ERK signaling and resulted in increases in cell growth, migration, and angiogenic ability, consequently endorsing the anti-proliferating effect of PP2A in endothelial cells [30]. An in vitro cell model study also found reduced PP2A activity and high levels of AKT and ERK phosphorylation in both primary TG (+) HEC cells and human HEC-P cells. This evidence supports our finding that ketamine-inhibited PDGF-BB-induced PI3K/Akt/ERK phosphorylation was significantly restored by both PP2A inhibitor okadaic acid and pp2a siRNA. Together, these results show that PP2A regulation plays a major role in ketamine’s inhibitory effect on VSMC proliferation.

PP2A activity is regulated by several molecular events, such as post-translation modification, auto-regulation, and substrate protein interaction. PP2A catalytic efficiency is reported to be controlled by two major alterations: phosphorylation [31] and methylation [32]. Tyrosine (Tyr) and leucine (Leu) undergo phosphorylation and methylation, respectively, and phosphorylation of Tyr307 successfully causes a decline in PP2A activity by preventing the interaction with PP2A-C [33]. Autophosphorylation-activated protein kinase is reported to inactivate the protein tyrosine phosphatase activity of PP2A [34]. PP2A-C expression is firmly controlled in the cell at the translational level, but not at the transcription level [35]. PP2A’s reversible methylation is a preserved controlling mechanism, and it has been reported that the methylation of Leu309 in a preserved TPDYFL motif in the C terminus of PP2A-c enhances holoenzyme assembly and phosphatase activity [36]. In addition to reversible methylation, PP2A-c has also been shown to endure tyrosine phosphorylation at Y307. Nevertheless, in contrast to methylation, tyrosine phosphorylation has been established to obstruct the catalytic function of PP2A [37]. In the present study, *pp2a* siRNA alone or combined with ketamine significantly inhibited the PDGF-BB-induced PP2A-C expression. Moreover, this study also found ketamine significantly and concentration-dependently suppressed the phosphorylation and demethylation of PP2A in VSMCs. These notable results further support our finding that ketamine inhibits PDGF-BB stimulated proliferation in VSMCs by the regulation of PP2A.

Our previous study had demonstrated that various concentrations of ketamine (100–500 μM) used to treated microglia did not cause cytotoxicity, and, at the higher concentration (500 μM), significantly inhibited TNF-α production [38]. Shibakawa et al. reported that ketamine at 100, 300, or 1000 µM significantly inhibited some of the inflammatory responses in microglial cells stimulated by lipopolysaccharide (LPS) without inducing cytotoxicity [39]. Moreover, it was reported that ketamine was used at up to 160 mg, which is equivalent to approximately 650 µM, in patients who had undergone retroperitoneal node dissection hemiscrotectomy surgery [40]. Therefore, we used this study a non-cytotoxic high concentration of 500 µM ketamine for its antiproliferative mechanistic effects in VSMCs.

## 3. Materials and Methods

### 3.1. Materials

The Dulbecco modified Eagle medium (DMEM), optiMEM, trypsin (0.25%), l-glutamine, penicillin, streptomycin, fetal bovine serum (FBS), 3-(4,5-dimethylthiazol-2-yl)-2,5-diphenyltetrazolium bromide (MTT), and dimethyl sulfoxide (DMSO) were purchased from Sigma-Aldrich (St. Louis, MO, USA). Okadaic acid was obtained from Calbiochem (San Diego, CA, USA), and 3-*O*-Methyl-sphingomyeline (3-OME-SM) was purchased from Biomol (Plymouth Meeting, PA, USA). The anti-phospho-p44/p42 extracellular signal-regulated kinase (ERK) (Thr^202^/Tyr^204^) was purchased from Cell Signaling (Beverly, MA, USA). The anti-phospho-Akt (Ser^473^) and anti-Akt monoclonal antibodies (mAbs) were purchased from Biovision (Mountain View, CA, USA). The anti-phospho-PP2A-C mAb, and anti-demethylated-PP2A-C mAb were purchased from Santa Cruz Biotechnology (Dallas, TX, USA). The anti-α-tubulin mAb was purchased from Neo Markers (Fremont, CA, USA). The horseradish peroxidase conjugated donkey anti-rabbit immunoglobulin G (IgG) and sheep anti-mouse IgG were purchased from Amersham (Buckinghamshire, UK). The Hybond-P polyvinylidene difluoride (PVDF) membrane and enhanced chemiluminescence (ECL) detection reagent were purchased from Amersham. Ketamine was dissolved in 0.1% DMSO, and stored at 4 °C.

### 3.2. Rat VSMC Primary Culture

The animal study was performed under protocols sanctioned by Taipei Medical University’s Animal Care and Use Committee (LAC-2015-0268, 28 December 2015). Male Wistar rats (250 to 300 g) procured from BioLASCO (Taipei, Taiwan), and maintained in agreement with standards published by the US National Institutes of Health (NIH Publication No. 85-23, revised 1996) following their Guide for the Care and Use of Laboratory Animals. To harvest VSMCs, thoracic aortas from Wistar rats were removed, and stripped of the endothelium and adventitia. The VSMCs were acquired by a modification of the combined collagenase and elastase digestion method [41]. The harvested VSMCs were grown in DMEM accompanied with 20 mM HEPES (4-(2-hydroxyethyl)-1-piperazineethanesulfonic acid), 10% FBS, 1% penicillin/streptomycin, and 2 mM l-glutamine at 37 °C in a humidified atmosphere of 5% CO_2_. The growth medium was changed every 2 to 3 days until cells were confluent. A trypsin-ethylenediaminetetraacetic (EDTA) solution was added, and the monolayer was incubated at 37 °C for 2 min. Containing 10 mL of DMEM, cells were detached and centrifuged at 900× *g* for 7 min before being resuspended in DMEM. For all the experiments, the cells from passages 4 to 8 were used. 

### 3.3. Cell Viability Assay

In 24-well plates, the VSMCs (2 × 10^4^ cells/well) were seeded and cultivated in DMEM containing 10% FBS for 24 h. VSMCs were pretreated with ketamine (50–500 µM) or solvent control (0.1% DMSO) for 30 min, followed by the addition of PDGF-BB (10 ng/mL) for 24 h. An MTT assay, which measures the mitochondrial activity, was used to determine cell viability [42]. The ratio of the absorbance of treated cells to that of the control cells (treated/control) multiplied by 100% was calculated as the cell number index. 

### 3.4. DNA Synthesis Assay

VSMCs (2 × 10^5^ cells/dish) were seeded in a 96-well microplate for 24 h and then serum-starved for 24 h. Following preincubation of ketamine (200 and 500 µM) or LY294002 (PI3K inhibitor, 30 μM) and PD98059 (ERK1/2 inhibitor, 20 μM) in serum-free medium for 30 min, the cells were treated with PDGF-BB (10 ng/mL) for 48 h. DNA synthesis was assessed using BrdU incorporation assay kits (Roche Diagnostics, Rotkreuz, Switzerland) according to the manufacturer’s instructions. DNA synthesis in VSMCs was assessed by the incorporation of BrdU. 

### 3.5. Immunoblot Analysis

Immunoblot analyses were executed as described previously [43]. Briefly, after the experimental periods, cells were lysed using an extraction buffer comprising 10 mM Tris (pH 7.0), 140 mM NaCl, 2 mM phenylmethylsulfonyl fluoride(PMSF), 5 mM DTT, 0.5% Nonidet P-40, 0.05 mM pepstatin A, and 0.2 mM leupeptin. Samples containing 50 µg of total protein were separated by SDS-PAGE using 12% polyacrylamide gels, and then electrotransferred onto PVDF membranes using a Bio-Rad semi dry transfer unit (Hercules, CA, USA). After blocking with Tris Buffered Saline with Tween (TBST, 10 mM Tris-base, 100 mM NaCl, and 0.01% Tween 20) containing 5% bovine serum albumin for 40 min, the blotted membranes were probed with various primary antibodies for 2 h. The membranes were incubated with horseradish peroxidase (HRP)-linked anti-mouse IgG or anti-rabbit IgG (diluted 1:3000 in TBST) for 1 h. The immunoreactive bands were pictured using enhanced chemiluminescent reagents (ECL, Amersham, Buckinghamshire, UK).

### 3.6. Suppression of PP2A Expression

As described previously [44], target gene suppression analysis was performed. A predesigned siRNA-targeting mouse *PP2A* gene was obtained from Ambion (Austin, TX, USA) for PP2A suppression analysis. The coding regions of the mouse PP2A catalytic subunit (*PP2A-C*) mRNA targeting the siRNA oligonucleotide were sequenced as *pp2a* siRNA, 5′-ccauacuccgagggaaucatt-3′. The negative control siRNA comprising a 19-bp scrambled sequence with 3d topicscape (3dT) overhangs was also purchased from Ambion.

### 3.7. Statistical Analysis

The results are presented as means ± standard errors from at least three independent experiments. The differences between the experiments were assessed using one-way analysis of variance (ANOVA). The statistical significance of the difference between means was determined using a Newman–Keuls test. A *p* value of <0.05 was considered statistically significant.

## 4. Conclusions

The present study showed that ketamine inhibited PDGF-BB-induced VSMC proliferation, which is critical in atherosclerosis. PP2A is an essential and miscellaneous phosphatase in the cellular system. An increasing body of evidence indicates that PP2A is known to regulate the activity of more than 30 different kinases, including PI3K, Akt, and ERK. In this study, we report the first preliminary evidence on the antiproliferative mechanism of action of ketamine in VSMCs. This inhibitory effect could be attributed to the PP2A-mediated inhibition of PI3K, Akt, and ERK, resulting in the overall inhibition of VSMC proliferation. Taken together, our findings support the notion that PP2A activation by ketamine may be an innovative means for therapeutic strategies to inhibit VSMC proliferation, as it plays a vital role in vascular diseases such as atherosclerosis and restenosis.
